# Engineering ‘cell robots’ for parallel and highly sensitive screening of biomolecules under *in vivo* conditions

**DOI:** 10.1038/s41598-017-15621-0

**Published:** 2017-11-09

**Authors:** Lifu Song, An-Ping Zeng

**Affiliations:** 1 Institute of Bioprocess and Biosystems Engineering, Hamburg University of Technology, Denickestrasse 15, D-21073 Hamburg, Germany; 2 Institute of Bioprocess and Biosystems Engineering, Hamburg University of Technology, Denickestrasse 15, D-21073 Hamburg, Germany

## Abstract

Cells are capable of rapid replication and performing tasks adaptively and ultra-sensitively and can be considered as cheap “biological-robots”. Here we propose to engineer cells for screening biomolecules in parallel and with high sensitivity. Specifically, we place the biomolecule variants (library) on the bacterial phage M13. We then design cells to screen the library based on cell-phage interactions mediated by a specific intracellular signal change caused by the biomolecule of interest. For proof of concept, we used intracellular lysine concentration in *E. coli* as a signal to successfully screen variants of functional aspartate kinase III (AK-III) under *in vivo* conditions, a key enzyme in L-lysine biosynthesis which is strictly inhibited by L-lysine. Comparative studies with flow cytometry method failed to distinguish the wild-type from lysine resistance variants of AK-III, confirming a higher sensitivity of the method. It opens up a new and effective way of *in vivo* high-throughput screening for functional molecules and can be easily implemented at low costs.

## Introduction

High-throughput screening (HTS) technologies are powerful tools with many successful applications, especially in directed evolution of biomolecules such as enzymes. They are primarily based on chemical or physical readouts such as fluorescence and assisted with miniaturized and/or parallel devices such as microfluidics and microchip, increasingly in an automated manner with the help of robotics^1–4^. These systems require expensive infrastructure and special expertise. The major focus was put on speeding up the screening process. For example, the state-of-the-art HTS technology based on fluorescence activated cell sorting (FACS) can reach 18,000–20,000 events per second^5^. However, signal detection with fast moving cells is a challenge which can result in noisy signals as shown by previous studies^6–9^. Furthermore, single cell variations are another source of signal noise which cannot be avoided by FACS based methods^10^. These represent some of the shortcomings of presently used HTS technologies when the molecules to be evolved and optimized are to be used for regulation and improvement of metabolic pathways in the context of metabolic engineering or for creating new synthetic pathways and regulation tools.

Similar to the electric robots, microbial cells can be considered as a kind of “biological robots” that can sense the information of fast changing environment, compute and make decisions for survival. Cells are highly programmable as proved by recent developments in synthetic biology. Programming cells to perform specific tasks have been achieved successfully in many cases. For example, cells have been programmed to produce pharmaceuticals, fuels, amino acids, fine and bulk chemicals and even metal nanoparticles^11–18^. Cells also have been programmed to sense toxic compounds in environments^19^, to record the environment signal in human gut^20^ and to eradicate human pathogen^21^. Although the capability of a single cell is limited, cells can reproduce themselves exponentially and work simultaneously to solve complicated tasks or accomplish sophisticated tasks in principle. However, these capabilities of cells have not yet been well exploited, especially for HTS purpose.

Recently, concentrations of intracellular molecules have been used as signals for overexpression of fluorescence for screening purposes in the context of strain improvement^8^. For example, Binder *et al*. successfully used the intracellular concentration of lysine, a natural lysine-responsive transcriptional activator LysG and fused expression of eYFP to screen high lysine producer from *C. glutamicum* ^7^. Later, by using the same sensor for *in vivo* detection of the desired end-product in single cells, they established a screening method with FACS to screen for enzymes without allosteric inhibition. However, due to the complexity of metabolic pathways, one enhanced enzyme usually has limited effects on productivity of the end-product. Genetic modifications are required to enhance the signal of the end-product in their studies^8^. Esvelt *et al*. (2011) presented an interesting phage-assisted method for continuous evolution of a specific gene-coded biomolecule that is linked to the infectivity of the phage mediated by the expression of a specific protein in host cells^22^. Specifically, M13 filamentous bacteriophage carrying the molecule of interest was used to infect *E. coli* cells in a lagoon with continuous inflow and outflow of the host cells, where the evolving gene is transferred from host cell to host cell in a manner that is dependent on the activity of the molecule of interest. The method was demonstrated with the evolution of a T7 RNA polymerase for new binding properties. It was later on used to successfully evolve proteases with significantly increased drug resistance to protease inhibitor^23–25^.

Here, we propose to use the cell-phage interactions mediated by the intracellular concentration of a specific metabolite for parallel and highly sensitive screening of biomolecules for metabolic pathway optimization under *in vivo* conditions. The basic idea is to program the cells to perform a certain screening task which is linked to the desired property or activity of the molecule of interest. The latter is in turn linked to the infectivity of the phage. Compared to physical robots the biological robots have the decisive advantage of fast replication, resulting in a large pool for simultaneously screening under *in vivo* conditions. Thus, the screening throughput can be expanded simply by using a larger population of cells, indicating a massively parallel screening manner potentially far beyond the current HTS technologies. It is also worth to mention that the cost for such an approach is almost zero compared to methods based on expensive FACS or microcapillary arrays, making it applicable in almost all biological labs.

We demonstrated the concept by screening mutants of a protein with reduced allosteric inhibition. Allosteric regulation is one of the fundamental mechanisms that control almost all cellular metabolism and gene regulation^26^. Deregulation of allosteric inhibition is essential in designing and optimizing metabolic pathways for the production of target metabolites such as amino acids^27^. Aspartate kinase III (AK-III), encoded by *lysC*, catalyzes the phosphorylation of aspartate and controls the biosynthesis of several industrially important amino acids such as lysine, threonine, and methionine in *E. coli* ^28^. AK-III is allosterically inhibited by L-lysine strictly. AK-III was chosen in this work as a model enzyme because of our extensive previous work on the rational design of it^27,29^. The new approach is shown to be more sensitive than the widely used flow cytometry method owing to a novel way of signal capturing.

## Results

### Principle of programming cells as robots for screening

**Figure 1:**
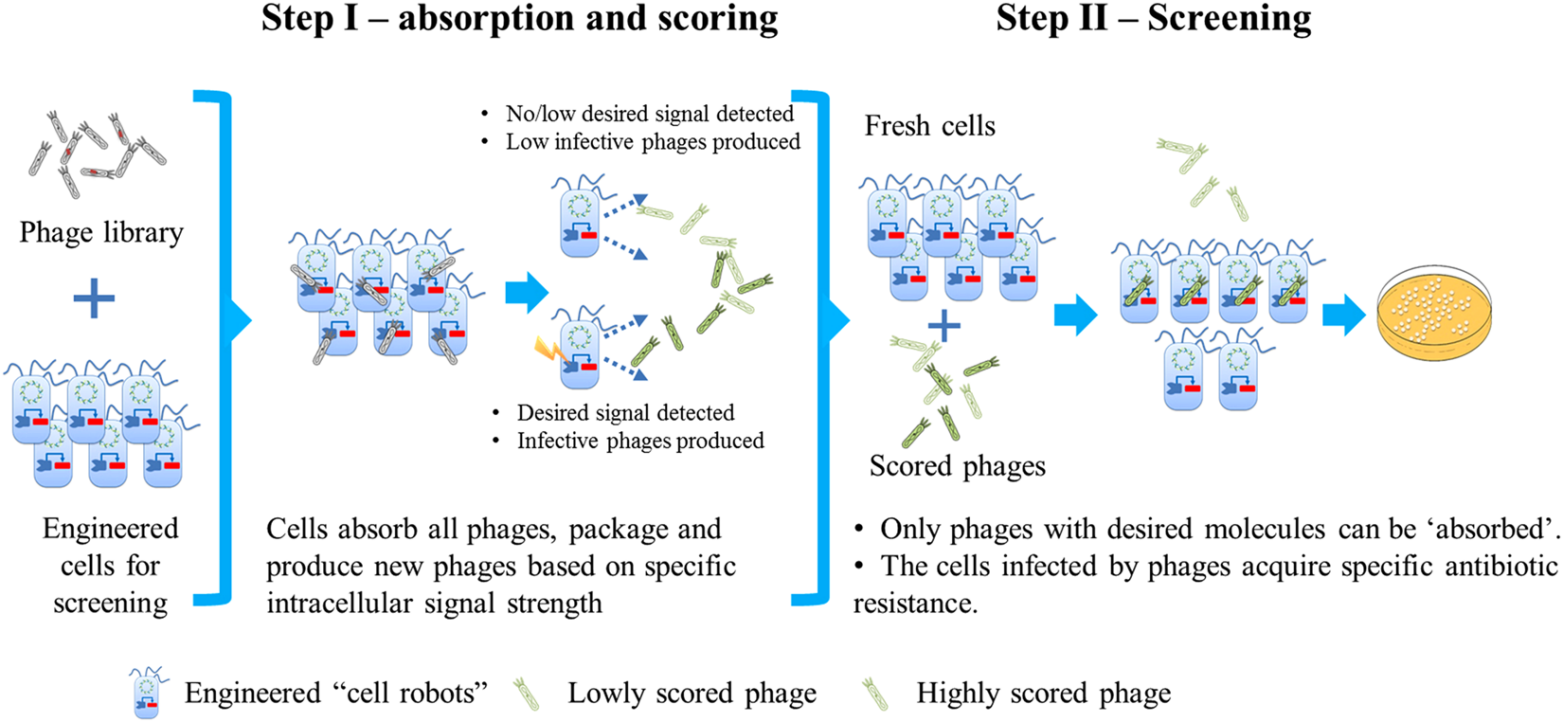
Work flow of cell robot based screening. A two-step screening strategy is suggested. First, phages are absorbed by engineered cells and packaged (scored) based on the performance of the molecules carried by the phages. Only the phages carrying molecules with desired properties are packaged in an effective way. Second, the ‘scored’ phages are absorbed by fresh host cells. In this step, only the infective phages, i.e. phages carrying molecules with the desired properties, are ‘absorbed’ by the host cells. The cells infected by the phages with desired molecules/targets acquire kanamycin resistance and can be easily identified by cultivation under kanamycin stress.

The workflow of programming cells as robots for the screening of molecule of interest (target) is shown in Fig. 1. Briefly, instead of placing the screening targets inside of the host cells as in most of the traditional screening methods, we place the targets to be screened on M13 phages. We then engineer the host cells so that they can screen for phages carrying the targets with desired properties. Specifically, we use *E. coli* XL1-Blue cells as the host cells for this purpose. To enable the host cells to control the infectivity of packaged phages, we transfer an essential gene for phage infectivity from the M13 phage to the host cells. The essential gene applied in this study is gene III encoding the attachment protein pIII which mediates adsorption of the phage to its primary receptor, the tip of *E. coli* F-pilus^30^. We then design an intracellular biological circuit to control the infectivity of packaged phages by controlling the expression level of gene III based on a specific intracellular signal that is related to the performance of the biomolecules to be screened, such as the concentration of an end product or an intermediate metabolite of a metabolic pathway. The targets are then cloned into VCSM13 by replacing the original gene III. A helper plasmid pJ175-Str which can supply the gene III product is used for preparing infective phage library at the first step of screening (see below). Elimination of gene III does not affect the phage secretion. However, the infectivity of the produced phages is very low. Thus, to enable an effective screening, we design a two-step strategy as illustrated in Fig. 1. In the first step, the phage library with the variants is ‘absorbed’ and ‘scored’ by the host cells based on the strength of the specific signal representing the performance of the target molecule. High-performance targets will produce more infectious phages than the low-performance ones. In the second step, the ‘scored’ phages are collected and screened in another run of cell–phage interaction. In this step, only infectious phages carrying the molecule with desired property can be ‘absorbed’. Since a kanamycin resistance gene (*aph*) is placed on the M13 phage, the cells capturing phages with desired properties can be selected easily by incubation under antibiotic pressure. In such a way, the target with the best performance under *in vivo* conditions can be effectively identified.

### Proof of concept of the method

**Figure 2:**
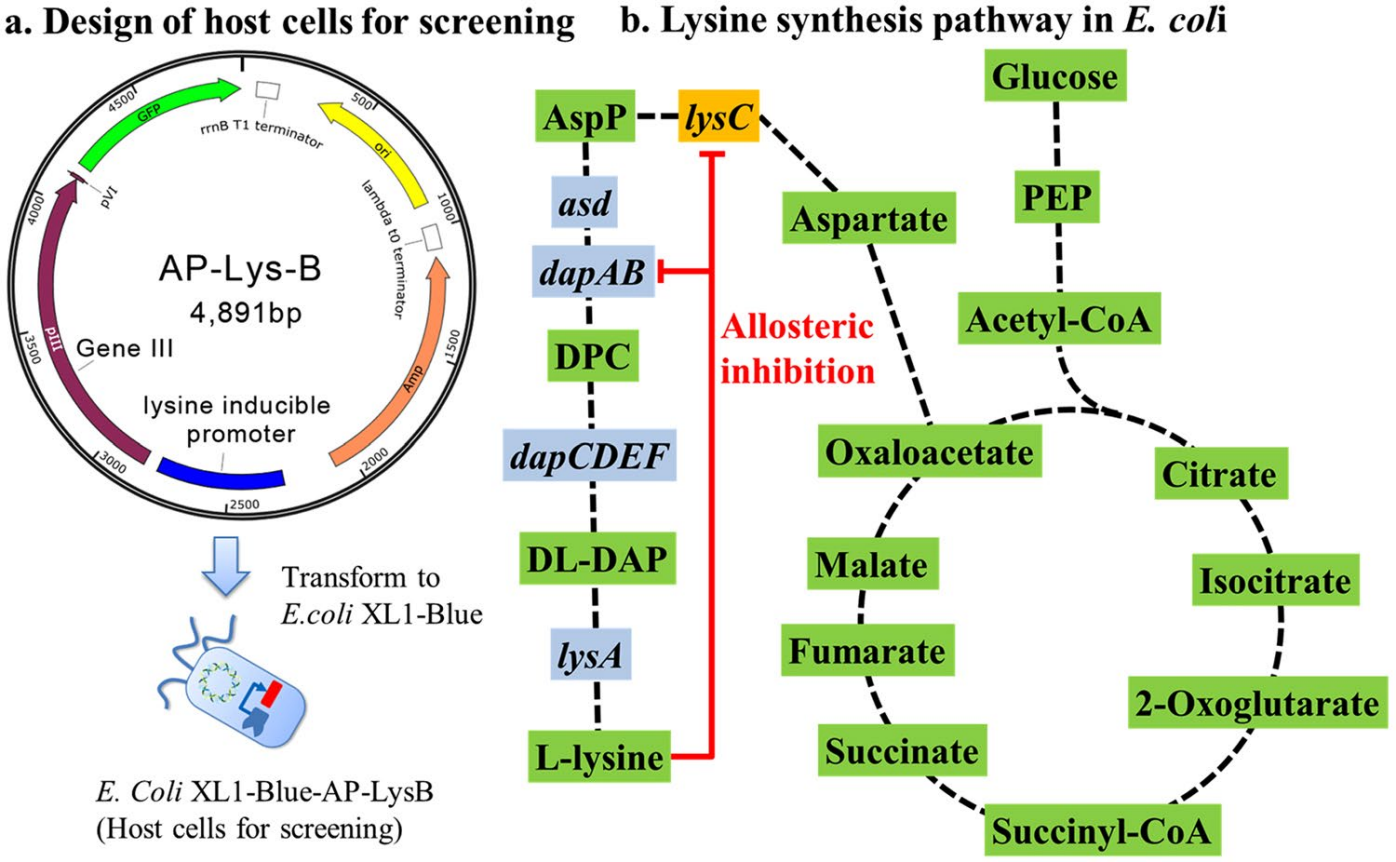
Illustration of designed “cell robots” screening for targets which can increase intracellular lysine concentration. (**a**) Plasmid map of the biological device controlling the scoring process based on intracellular lysine concentration. A lysine inducible promoter was cloned from *C. glutamicum* ATCC13032 as lysine sensor. The gene III of M13 phage and a green fluorescence protein (GFP) encoding gene were placed under the control of the lysine inducible promoter. The GFP-encoding gene was used for comparing the sensitivity of the cell-phage based screening with the flow cytometry-based screening. It is not required for the cell robot based screening. (**b**) Biosynthesis pathway of lysine in *E. coli*. Green rectangles represent metabolites, light blue and yellow rectangles the names of related genes. In principle, the engineered host cells can be used to screen any enzymes in the lysine biosynthesis pathway for enhanced enzyme performance. In the proof of concept study, we focused on screening mutants of AK-III (encoded by the *lysC* gene) with reduced allosteric inhibition by lysine. PEP – phosphoenolpyruvate; AspP – L-aspartyl-4-phosphate; DPC – Tetrahydrodipicolinate; DL-DAP – D,L-diaminopimelate.

To use intracellular lysine concentration as a signal for the screening of an L-lysine-resistant aspartate kinase (AK-III, encoded by *lysC* gene in *E. coli*) based on the cell-phage interaction, a lysine inducible promoter was cloned from *Corynebacterium glutamicum* ATCC13032 as the lysine sensor^31^. The gene III from M13 phage was cloned into the plasmid AP-Lys-B and placed under the control of the lysine inducible promoter (Fig. 2a). The screening “cell robots”, namely *E. coli* XL-Blue-AP-Lys-B, were then obtained by transforming *E. coli* XL-Blue cells with the plasmid AP-Lys-B. In such a way, if a phage carrying a target gene that can increase the intracellular lysine concentration is absorbed by the *E. coli* cells, it will increase the gene III expression level, which will result in the production of infective phages. Thus, the desired targets which can increase intracellular lysine concentration can be identified by using the two-step screening strategy.

To experimentally demonstrate the functioning of the method, the wild-type *lysC* gene was first cloned from *E. coli* MG1655 to VCSM13 phage by replacing the Gene III. The obtained phagemid is named as M13-lysC. We then constructed another phagemid M13-lysC-V339A by introducing a site mutation to the *lysC* gene of M13-lysC. The V339A mutant of AK-III has been previously proven to be well resistant to allosteric inhibition by lysine^27^. We mixed roughly equal amount phage particles of M13-lysC and M13-lysC-V339A and screened the mixture of the phages using the designed host cells. If the screening robots function as expected, the phages of M13-lysC-V339A should be screened out. We repeated the experiments three times with the designed host cells using lysine as signals for screening. One time we used cells cultivated with LB medium and twice with cells cultivated with M9 medium, representing differential expression levels of lysine synthesis pathway genes under various conditions. To verify the genotypes of the resulting phages, plasmids extracted from six individual colonies were sequenced for each experiment. All colonies were verified to be M13-lysC-V339A in all three experiments, confirming a robust screening function of the designed “cell robots”.

**Figure 3:**
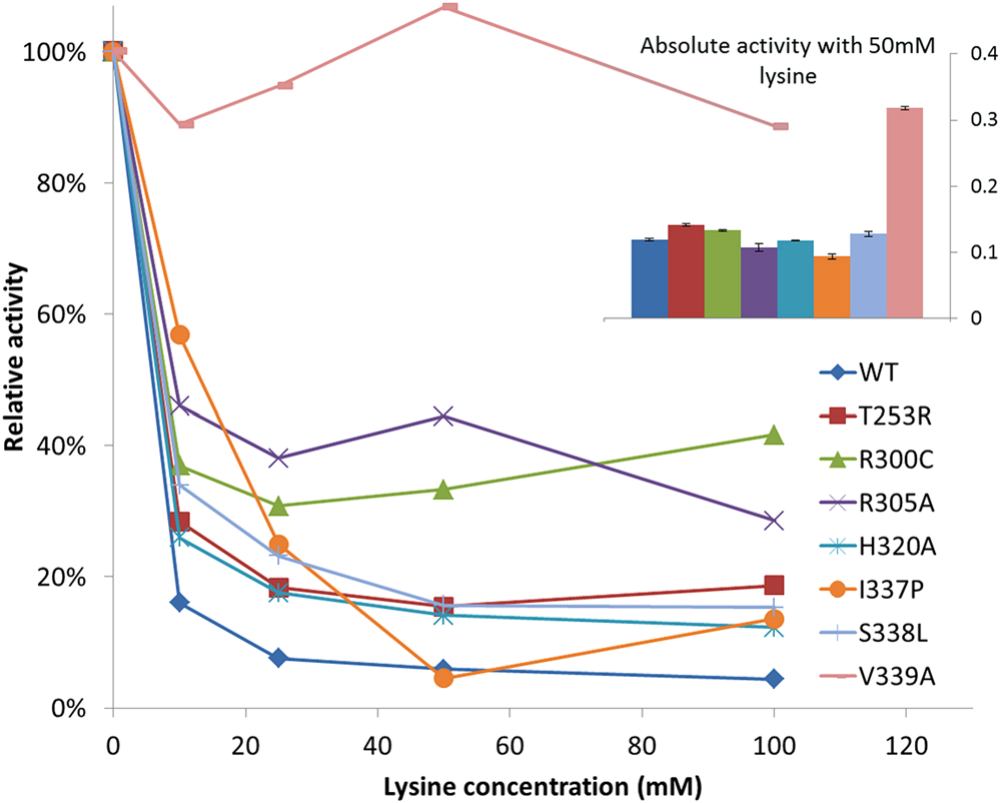
Inhibition profiles of wild-type and muteins of AK-III. *In vitro* enzyme assays were performed to characterize the inhibition profiles of wild-type and mutants of AK-III by lysine. The activities were displayed as relative activities normalized by the specific activities without lysine inhibition. The specific activities with 50 mM lysine presented by normalized absorbance by protein concentration are shown by the small histogram top-right. Data represent mean values and standard deviation from three assays.

In a further experiment, we screened a variant pool containing the wild-type AK-III, six AK-III mutants from previous study^27^ and a new mutant (R300C) obtained from screening a relatively small size of library with the present method (data not shown). In the first run of screening, we selected 12 colonies for sequencing which gave the following results: 5 for the variant V339A, 3 for the variant I337P, 2 for the variant S338L, and 1 for each of the variant T253R and H320A. In the second run using the phage mix from the 1^st^ screening, we obtained only one colony of V339A. As shown in Fig. 3, the variant V339A has the highest activity and resistance against lysine, confirming the effectiveness of the two-step approach.

### Screening with a higher sensitivity than fluorescence-based method

**Figure 4:**
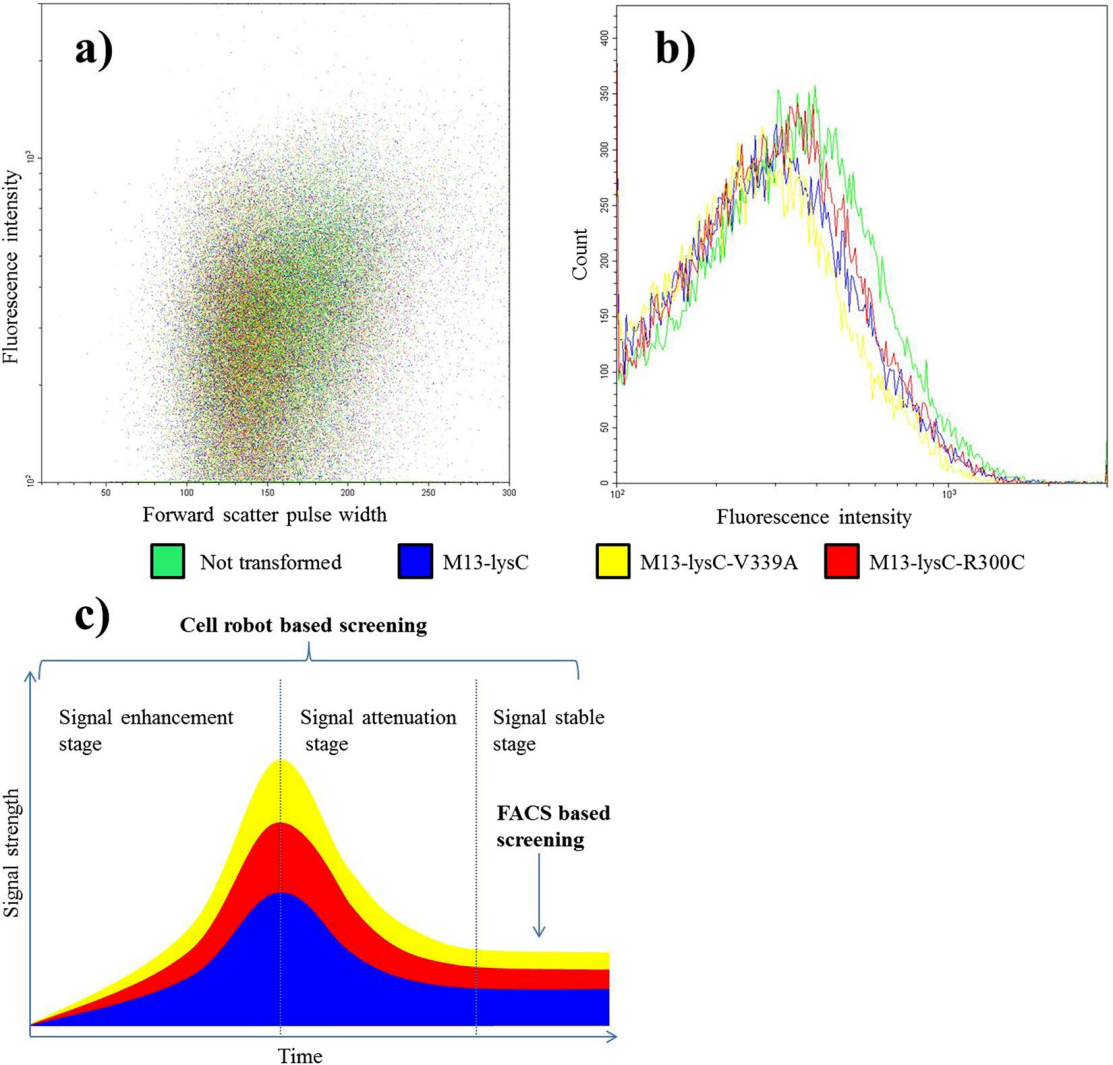
Flow Cytometry assays of cell populations harboring wild-type AK-III and AK-III muteins. (**a**) Dot plot of flow cytometry assay results. (**b**) Statistic analysis of the total cell numbers at different fluorescence intensities. (**c**) Illustration of differences in signal capture of the cell-robot based and the FACS-based screening methods. Phagemids M13-lysC, M13-lysC-V339A and M13-lysC-R300C were transformed to *E. coli* XL1-Blue/AP-Lys-B cells individually. Flow Cytometry assays were performed on the obtained cell populations by measuring green fluorescence intensity. Although slight differences could be observed in figure (**b**), the flow cytometry method failed to distinguish the mutants despite varying the gain setting. Introduction of molecular variants into cells can be regarded as perturbations to the cells. Figure c illustrates possible change of signal after introduction of variants (perturbations): enhancement, attenuation and stabilization.

To compare the sensitivity of the current screening system with methods based on fluorescence and flow cytometry, the state-of-the-art screening technology, we transformed *E. coli* XL1-Blue/AP-Lys-B cells with M13-lysC, M13-lysC-V339A, and M13-lysC-R300C individually to obtain cells of XL1-Blue/AP-Lys-B/M13-lysC-WT, XL1-Blue/AP-Lys-B/M13-lysC-V339A, and XL1-Blue/AP-Lys-B/M13-lysC-R300C. A GFP-encoding gene was placed under the control of the same lysine sensing sensor in *E. coli* XL1-Blue/AP-Lys-B. Over-night cultivated cells of XL1-Blue/AP-Lys-B, XL1-Blue/AP-Lys-B/M13-lysC-WT, XL1-Blue/AP-Lys-B/M13-lysC-V339A and XL1-Blue/AP-Lys-B/M13-lysC-R300C were harvested and washed twice with 50 mM, ice-cooled PBS buffer. The fluorescence activities of the four different cell populations were measured using flow cytometry. As shown in Fig. 4b, although slight differences could be observed, it was not possible to set up a gain setting to select the mutants. In other words, the different cell populations cannot be distinguished by the flow cytometry method. On the other hand, our method based on cell-phage interaction can successfully screen out V339A as the best mutant of AK-III as shown in Fig. 3, confirming a higher sensitivity of the cell robot based screening method.

Biological systems are complex and highly adaptive, meaning that the cells always try to reduce the perturbations introduced. Introducing molecular variants into cells can be regarded as perturbations to the cells. As illustrated in Fig. 4c, after the introduction of molecular variants, the cells may undergo three stages of signal change: enhancement, attenuation and stabilization. The signal enhancement stage is the direct consequence of the perturbations induced by the introduced molecular variants. The signal attenuation stage is caused by adaptive response of cells to the perturbation. Finally, the signal reaches a stable state which might be slightly different from the state before the perturbation. The time interval for these changes may be relatively short. The curves in Fig. 4c are theoretical response patterns of cells to the disturbance by over-expression of the different AK-III variants respectively. For the FACS based method, the cells to be measured may have already reached the stable stage where the signal strength may not be significantly different in the cell populations with different variants. However, our method captures signals during the whole response and adaptation processes which correspond to the area below the curves and can be therefore more sensitive.

Furthermore, the “cell robots” based screening works in principle like an autocatalytic process of signal amplification: the target molecule with desired performance will increase the intracellular concentration of the signal molecule (in this case lysine) in the cell, the increased concentration of the signal molecule will amplify the population of phage carrying the target molecule. The amplified phages can infect other cells to further enhance the signal. In such a way the screening process is highly effective and sensitive compared to the other presently used methods, such as those based on single cells using fluorescence as readout signal^8^.

## Discussion

In this study, we demonstrated that the biological ‘robots’, i.e. the cells, can be engineered to perform screening tasks in protein engineering. By capturing the signals during the whole response and adaptation processes, which cannot be achieved by screening based on electric machines, the cell-phage based screening system has an inherent higher sensitivity. The current proof of concept study shows that cell-phage interaction system does not require any genetic modifications of the host cells to enhance the signal for screening. In a recent similar work which used FACS as the screening method, genetic modifications are required to enhance the signal^8^. Furthermore, FACS based screening suffers often signal noise caused by single cell variations and signal detection under conditions of fast moving cells^7,8,10^. By equally accessing all cells, the cell-phage interaction system can avoid the problem of single cell variation in principle. Cells as biological ‘robots’ have a unique feature of reproducing themselves to generate a vast population exponentially and cheaply. Thus, the screening throughput can be expanded simply by using a larger population of cells with minimal additional costs, indicating a massively parallel screening manner beyond the current electric based machines. As proved by the power of parallel computing in computational science^32^, parallelization is a great solution for speeding up the process of parallel tasks. The sensitivity and throughput are key factors determining the success of a screening experiment. The cell-phage screening system shows clear advantages in both sensitivity and throughput. Furthermore, the cost of cell robots is almost zero compared to that of expensive electric machines/robots. It should be mentioned that, while electric machines can utilize various types of signals for screening, screening based on the cell robots uses a “biological signal and sensor”, which might represent a limitation in some cases. However, many natural or purposefully designed biological elements or sensors such as promoters and riboswitches can be used for this purpose^33,34^ and the signal molecules can be intermediates of metabolic pathways.

## Materials and Methods

### Strains, phages and plasmids

The M13 phage (VCSM13) was purchased from Agilent Technology (5301 Stevens Creek Blvd. Santa Clara, CA 95051, USA). The wild *lysC* gene encoding AK-III was amplified by PCR from the genomic DNA of *E. coli* K12 MG1655. For over-expression and purification of the wild-type AK-III and relevant muteins, the wild-type *lysC* gene was cloned to pET-22b(+) with the introduction of an additional His-tag at the C-terminal to generate the plasmid pET22-lysC. Site-mutagenesis was performed on pET22-lysC to generate over-expression plasmids for AK-III muteins. The *lysC* gene was also cloned to VCSM13 by replacing the original gene III to generate a phagemid M13-lysC. Similarly, site-mutagenesis was also performed on M13-lysC to generate phagemid derivations carrying different AK-III muteins.

For construction of plasmid AP-Lys-B, i.e. the device harnessed by the host cells to control the phage packaging process based on intracellular lysince concentration, we ultilized a lysine inducible promoter from *Corynebacterium glutamicum* ATCC13032 as a lysine sensor^31^. The lysine inducible promoter, gene III from M13 phage and a GFP-encoding gene were cloned into the plasmid pZE21MCS to obtain AP-Lys-B as shown in Fig. 2a. The transcriptional levels of gene III and GFP encoding gene are controlled by the lysine inducible promoter. The antibiotic resistance type of AP-Lys-B was changed to ampicillin resistance by replacing the kanamycin resistance gene with an ampicillin resistance gene. All used strains, plasmids in current study are listed in Table 1.

**Table 1:** Strains, plasmids and primers used in this study.

Strains/phages/plasmids/primers	Description/Genotype	Note
Strains		
*E. coli* XL1-Blue		Agilent
*E. coli* XL1-Blue/pJ175e	*E. coli* XL1-Blue harboring pJ175e	
*E. coli* XL1-Blue/AP-Lys-B	*E. coli* XL1-Blue harboring AP-Lys-B	
*E. coli* XL1-Blue/AP-Lys-B /M13-lysC	*E. coli* XL1-Blue harboring AP-Lys-B and M13-lysC	
*E. coli* XL1-Blue/AP-Lys-B /M13-lysC-V339A	*E. coli* XL1-Blue harboring AP-Lys-B and M13-lysC-V339A	
*E. coli* XL1-Blue/AP-Lys-B /M13-lysC-R300C	*E. coli* XL1-Blue harboring AP-Lys-B and M13-lysC-R300C	
Phages		
VCSM13	*Kan*	Agilent
M13-lysC	Derived from VCSM13 by replacing gene III with wild *lysC* from *E. coli* K12	Plasmid map and full sequence are detailed in Supplementary information.
M13-lysC-T253R	Derived from M13-lysC by site mutagenesis	
M13-lysC-R300C	Obtained by screening with a library of M13-lysC generated by *in vivo* random mutagenesis	
M13-lysC-R305A	Derived from M13-lysC by site mutagenesis	
M13-lysC-H320A	Derived from M13-lysC by site mutagenesis	
M13-lysC-I337P	Derived from M13-lysC by site mutagenesis	
M13-lysC-S338L	Derived from M13-lysC by site mutagenesis	
M13-lysC-V339A	Derived from M13-lysC by site mutagenesis	
Plasmids		
pJ175e	*Amp*, obtained from David Group	
AP-Lys-B	*Amp*, Derived from pZE21 plasmid;	Plasmid map and full sequence are detailed in Supplementary information.
pET22-lysC	*Amp*, Expression plasmid for wild-type AK-III	
pET22-lysC-T253R	Expression plasmid for T253R mutant of AK-III	
pET22-lysC-R300C	Expression plasmid for R300C mutant of AK-III	
pET22-lysC-R305A	Expression plasmid for R305A mutant of AK-III	
pET22-lysC-H320A	Expression plasmid for H320A mutant of AK-III	
pET22-lysC-I337P	Expression plasmid for I337P mutant of AK-III	
pET22-lysC-S338L	Expression plasmid for S338L mutant of AK-III	
pET22-lysC-V339A	Expression plasmid for V339A mutant of AK-III	
Primers	Description	Sequence (5′−3′)
M13Seq-G3-P1	Sequencing primer	TCTGTAGCCGTTGCTACCCTCGTT
M13Seq-G3-P2	Sequencing primer	AAGAAACAATGAAATAGCAATA
M13-ln4Genes-P1	Primer for linearization of VCSM13	CTAGTATTTCTCCTCTTTCTCTAGTATAATTGTATCGGTTTATCAGCTTGCT
M13-ln4Genes-P2	Primer for linearization of VCSM13	CTCCCTCAATCGGTTGAATGT
LysC-4M13-P1	For cloning of *lysC*	GAGGAGAAATACTAGATGTCTGAAATTGTTGTCTCC
LysC-4M13-P2	For cloning of *lysC*	AACCGATTGAGGGAGTTACTCAAACAAATTACTATG
V339A-P1	Site-directed mutagenesis of *lysC* to generate V339A mutant	GCAGACTTAATCACCACGTCAGAA G
V339A-P2	Site-directed mutagenesis of *lysC* to generate V339A mutant	CGAAATATTATGCCGCGCGAGGAT G
T253R-P1	Site-directed mutagenesis of *lysC* to generate T253R mutant	CGTTTTGGTGCAAAAGTACTGC
T253R-P2	Site-directed mutagenesis of *lysC* to generate T253R mutant	TGCCATCTCTGCCGCTTCGGCA
R305A-P1	Site-directed mutagenesis of *lysC* to generate R305A mutant	TGCTCGCAATCAGACTCTGCTC
R305A-P2	Site-directed mutagenesis of *lysC* to generate R305A mutant	AGCGCCAGAGCGCGGAACAGCG
H320A-P1	Site-directed mutagenesis of *lysC* to generate H320A mutant	TTCTCGCGGTTTCCTCGCGGAA
H320A-P2	Site-directed mutagenesis of *lysC* to generate H320A mutant	GCCAGCATATTCAGGCTGTGCA
I337P-P1	Site-directed mutagenesis of *lysC* to generate I337P mutant	CTTCGGTAGACTTAATCACCAC
I337P-P2	Site-directed mutagenesis of *lysC* to generate I337P mutant	GATTATGCCGCGCGAGGATGCC
S338L-P1	Site-directed mutagenesis of *lysC* to generate S338L mutant	TGGTAGACTTAATCACCACGTC
S338L-P2	Site-directed mutagenesis of *lysC* to generate S338L mutant	AAATATTATGCCGCGCGAGGAT
R300C-P1	Site-directed mutagenesis of *lysC* to generate R300C mutant	TGCGCTCTGGCGCTTCGTCGCAATC
R300C-P2	Site-directed mutagenesis of *lysC* to generate R300C mutant	GAACAGCGGCGGATTTTCAGTTTTA

### Molecular cloning

All PCR experiments for cloning were performed using Thermo Scientific PCR Master Mix or CloneAmp HiFi PCR Premix. Cloning experiments were performed using In-Fusion HD Cloning Plus. Site-mutagenesis was performed using a protocol similar to the NEB Q5® Site-Directed Mutagenesis Kit. Briefly, none overlap primers were designed and synthesized which contain the desired mutations. Then PCR amplification was performed with the designed primers using the original plasmid as templates to generate linear plasmids. Template DNA was eliminated by enzymatic digestion with DpnI. Finally, phosphorylation and ligation using T4 Polynucleotide Kinase and T4 Ligase were carried out to obtain circular DNA before transformation.

### Mutagenesis

Random *in vivo* mutagenesis was enabled by using the plasmid pJ184-Str harboring genes which can increase intracellular DNA replication error rates. The plasmid pJ184-Str was derived from pJ184 by replacing the chloramphenicol acetyltransferase encoding gene with a streptomycin resistance gene. The pJ184 plasmid which has been described previously was obtained from David R. Liu’s group of Harvard Medical School^22^.

### Preparation of infective phages for screening

Since the engineered phages lack gene III, the helper plasmid pJ175e was harnessed by the host cells to supply gene III products intracellularly to obtain infective phages. The plasmid pJ175e was obtained from David R. Liu’s group. Specifically, engineered phages were co-transformed with pJ175e into XL1-Blue cells. Overnight cultures were deposited for centrifuge and the supernatant containing the packaged infective phages was collected.

### Screening based on cell robots

XL1-Blue/AP-Lys-B cells, i.e. the “cell robots”, were incubated in LB medium to an OD_600_ value around 1.0. Roughly 200ul XL1-Blue/AP-Lys-B cells were mixed with 2ul proper diluted phages (Cells to phage number ratio above 10:1 to make sure that all phages could be captured and evaluated by host cells. Different types of phages in a total number of roughly 10,000 were used as inputs in the present study). The mixture was incubated at 37 °C for 15 minutes without shaking to allow the phages to attach to the cells, following by incubation at 37 °C with shaking for 1 to 2 hours. Inactivate the host cells at 65 °C for 15 min. The cell debris were spinned down and the supernatant containing the “scored” phages was transferred to a fresh tube. A proper amount of “scored” phages were mixed with fresh XL1-Blue/AP-Lys-B cells and incubated at 37 °C for 15 minutes without shaking to allow the “cell robots” to absorb the highly “scored” phages. A proper amount of the culture was then sprayed on LB agar plates with kanamycin (50 mg/ml) for screening.

### Protein purification and enzyme activity assay

Enzymes were expressed in *E. coli* BL21 (DE3) cells (New England Biolabs, Germany) using pET derived plasmids. The recombinant cells were first cultivated in LB media supplemented with 50 μg/mL kanamycin at 37 °C to reach an OD600 of 0.6 and then protein expression was induced by adding 0.1 mM isopropyl β-D-thiogalactopyranoside (IPTG) for overnight at 30 °C. The harvested cells were washed twice with 20 mM Tris-HCl buffer (pH 7.0) and suspended in a buffer of 50 mM Na_2_HPO_4_ (pH 7.0), 0.2 mM EDTA and 0.1 mM dithiothreitol. Suspended cells were disrupted and centrifuged at 100,000 g for 1 h. The crude enzymes, e.g. the supernatant, was purified using a Ni_2_^+^-NTA column (GE Healthcare Bio-Sciences, Piscataway, NJ) to obtain samples for activity assay.

The aspartokinase activities of AK-III and muteins were assayed using the hydroxamate method^35^. In details, 1 ml reaction mixture contained 200 mM Tris–HCl (pH 7.5), 10 mM MgSO_4_·6H_2_O, 10 mM aspartate, 10 mM ATP, 160 mM NH_2_OH·HCl and appropriate amounts of enzyme. After incubation at 30 °C for 30 min, the reaction was stopped by mixing with 1 ml 5% (w/v) FeCl_3_ solution and the absorbance at 540 nm was monitored.

### Flow cytometry analysis

Overnight cultured cells were washed and re-suspended in ice-cooled 50 mM PBS buffer and diluted to a cell density of approximately 3 × 10^6^ cells/ml. Then, cellular GFP was analyzed by using flow cytometry (Beckman Coulter CytoFlex) with excitation at 488 nm and detecting fluorescence at 529 ± 14 nm. For each sample, 50,000 events were recorded. Data were analyzed using the CytExpert software.

### Data availability statement

All data are provided in full in the results section of this paper.

## Electronic supplementary material

Plasmid map and full sequences of M13-lysC and AP-Lys-B
