# Supplementary material for: Engineering ‘cell robots’ for parallel and highly sensitive screening of biomolecules under in vivo conditions
Source: Sci Rep. 2017 Nov 9;7:15145. doi: 10.1038/s41598-017-15621-0 (PMC5680304; doi:10.1038/s41598-017-15621-0)
Supplement: Supplementary file 1 — Plasmid map and full sequences of M13-lysC and AP-Lys-B [file 41598_2017_15621_MOESM1_ESM.pdf]

Engineering 'cell robots' for parallel and highly sensitive screening of biomolecules under *in vivo* conditions

Lifu Song[1] and An-Ping Zeng[2*]

[1] Institute of Bioprocess and Biosystems Engineering, Hamburg University of Technology, Denickestrasse 15, D-21073 Hamburg, Germany. E-mail: lifu.ibb@gmail.com.

[2] Institute of Bioprocess and Biosystems Engineering, Hamburg University of Technology, Denickestrasse 15, D-21073 Hamburg, Germany. Phone: +49 (0)40 42878 – 4183. E-mail: aze@tuhh.de.

*corresponding author

Supplementary information

Plasmid map and full sequences of M13-lysC and AP-Lys-B.

Plasmid maps of AP-lys-B

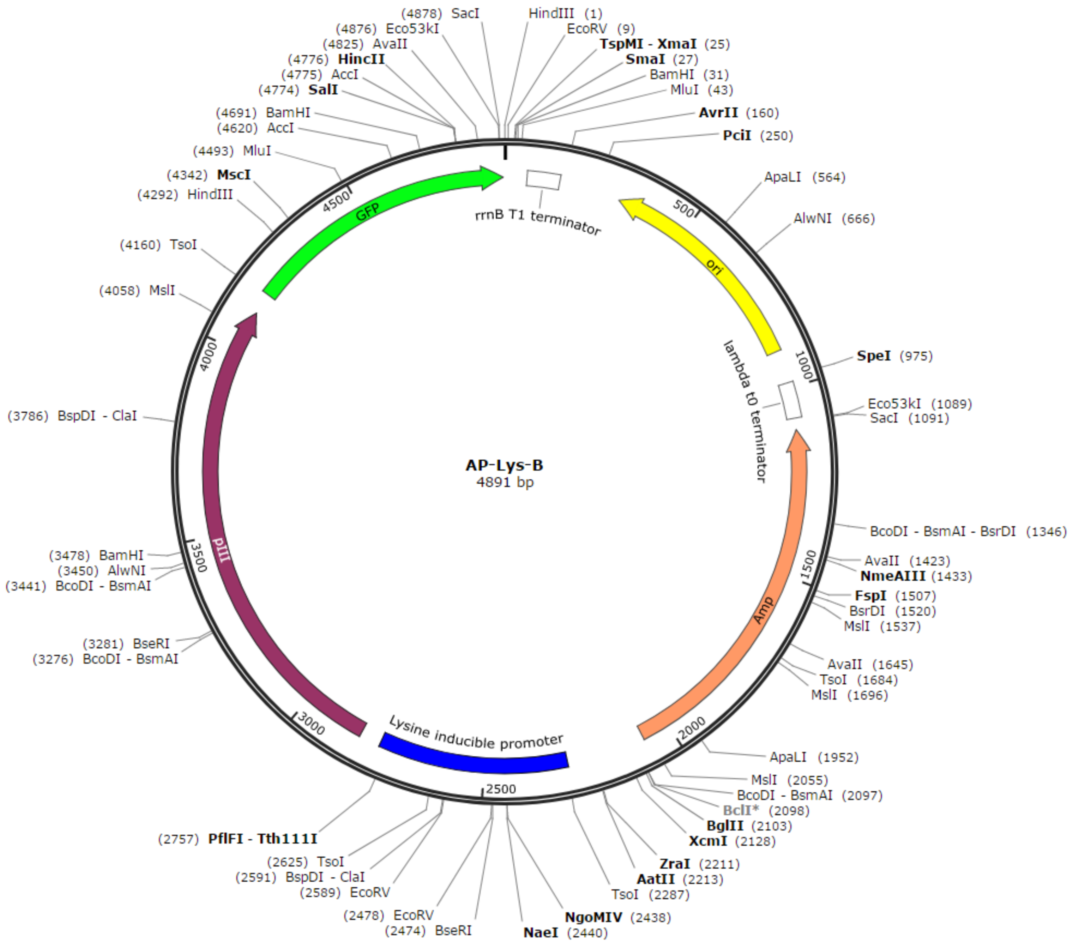

Full sequences of AP-lys-B

AAGCTTGATATCGAATTCCTGCAGCCCGGGGGATCCCATGGTACGCGTGCTAGAGGCATCAAATAAAACGAA
AGGCTCAGTCGAAAGACTGGGCCTTTCGTTTTATCTGTTGTTTGTCGGTGAACGCTCTCCTGAGTAGGACAA
ATCCGCCGCCCTAGACCTAGGGCGTTCGGCTGCGGCGAGCGGTATCAGCTCACTCAAAGGCGGTAATACGG
TTATCCACAGAATCAGGGGATAACGCAGGAAAGAACATGTGAGCAAAAGGCCAGCAAAAGGCCAGGAACC
GTAAAAAGGCCGCGTTGCTGGCGTTTTTCCATAGGCTCCGCCCCCCTGACGAGCATCACAAAAATCGACGCT
CAAGTCAGAGGTGGCGAAACCCGACAGGACTATAAAGATACCAGGCGTTTCCCCCTGGAAGCTCCCTCGTG
CGCTCTCCTGTTCCGACCCTGCCGCTTACCGGATACCTGTCCGCCTTTCTCCCTTCGGGAAGCGTGGCGCTTT
CTCAATGCTCACGCTGTAGGTATCTCAGTTCGGTGTAGGTCGTTCGCTCCAAGCTGGGCTGTGTGCACGAAC
CCCCCGTTCAGCCCGACCGCTGCGCCTTATCCGGTAACTATCGTCTTGAGTCCAACCCGGTAAGACACGACTT
ATCGCCACTGGCAGCAGCCACTGGTAACAGGATTAGCAGAGCGAGGTATGTAGGCGGTGCTACAGAGTTCT
TGAAGTGGTGGCCTAACTACGGCTACACTAGAAGGACAGTATTTGGTATCTGCGCTCTGCTGAAGCCAGTTA
CCTTCGGAAAAAGAGTTGGTAGCTCTTGATCCGGCAAACAAACCACCGCTGGTAGCGGTGGTTTTTTTGTTT
GCAAGCAGCAGATTACGCGCAGAAAAAAAGGATCTCAAGAAGATCCTTTGATCTTTTCTACGGGGTCTGAC
GCTCAGTGGAACGAAAACTCACGTTAAGGGATTTTGGTCATGACTAGTGCTTGGATTCTCACCAATAAAAAA

CGCCCGGCGGCAACCGAGCGTTCTGAACAAATCCAGATGGAGTTCTGAGGTCATTACTGGATCTATCAACA
GGAGTCCAAGCGAGCTCTCGAACCCCAGAGTCCCGCtcatgagattatcaaaaaggatcttcacctagatccttttaaatta
aaaatgaagttttaaatcaatctaaagtatatatgagtaaacttggtctgacagttaccaatgcttaatcagtgaggcacctatctcagcgatc
tgtctatttcgttcatccatagttgcctgactccccgtcgtgtagataactacgatacgggagggcttaccatctggccccagtgctgcaatgat
accgcgagacccacgctcaccggctccagatttatcagcaataaaccagccagccggaagggccgagcgcagaagtggtcctgcaacttta
tccgcctccatccagtctattaattgttgccgggaagctagagtaagtagttcgccagttaatagtttgcgcaacgttgttgccattgctacagg
catcgtggtgtcacgctcgtcgtttggtatggcttcattcagctccggttcccaacgatcaaggcgagttacatgatcccccatgttgtgcaaaa
aagcggttagctccttcggtcctccgatcgttgtcagaagtaagttggccgcagtgttatcactcatggttatggcagcactgcataattctctt
actgtcatgccatccgtaagatgcttttctgtgactggtgagtactcaaccaagtcattctgagaatagtgtatgcggcgaccgagttgctcttg
cccggcgtcaatacgggataataccgcgccacatagcagaactttaaaagtgctcatcattggaaaacgttcttcggggcgaaaactctcaa
ggatcttaccgctgttgagatccagttcgatgtaacccactcgtgcacccaactgatcttcagcatcttttactttcaccagcgtttctgggtgag
caaaaacaggaaggcaaaatgccgcaaaaaagggaataagggcgacacggaaatgttgaatactcatGCGAAACGATCCTCATC
CTGTCTCTTGATCAGATCTTGATCCCCTGCGCCATCAGATCCTTGGCGGCAAGAAAGCCATCCAGTTTACTTT
GCAGGGCTTCCCAACCTTACCAGAGGGCGCCCCAGCTGGCAATTCCGACGTCTAAGAAACCATTATTATCAT
GACATTAACCTATAAAAATAGGCGTATCACGAGGCCCTTTCGTCTTttgcctctagtgggactggctatgaaccgaacgca
gtgccggactacatccgatgattttctcattgatcccctgcatatcactgacggtgatgtaggcgaggaaatcgaagcatatctagtgggtgcc
tactgcatcgaagatgagctgattttacgccggcgaatccgcttcccgagaggagtcaaaccaggagatatcatcggaattcctaacaccgc
aggatacttcatgcatatcttggaaagtgcatcgcaccaaatcccgttggcgaaaaatgtagtgtggccggaggggcagttagacgatatcg
atgcggattaagacataaccattcgctaatctttcgacgccccttctgaggtggggatttcttttcatcccctttaattattttcggaaatttatac
agcaatcctcgaaatcctaataaagatcccttatcgtgggagaggtacggtagttcgttcgaggacaacgtcgagaaaggcatgatttcaTa
aaccgatacaattaaaggctccttttggagcctttttttttggagattttcaacgtgaaaaaattattattcgcaattcctttagttgttcctttcta
ttctcactccgctgaaactgttgaaagttgtttagcaaaaccccatacagaaaattcatttactaacgtctggaaagacgacaaaactttagat
cgttacgctaactatgagggctgtctgtggaatgctacaggcgttgtagtttgtactggtgacgaaactcagtgttacggtacatgggttcctat
tgggcttgctatccctgaaaatgagggtggtggctctgagggtggcggttctgagggtggcggttctgagggtggcggtactaaacctcctg
agtacggtgatacacctattccgggctatacttatatcaaccctctcgacggcacttatccgcctggtactgagcaaaaccccgctaatcctaa
tccttctcttgaggagtctcagcctcttaatactttcatgtttcagaataataggttccgaaataggcagggggcattaactgtttatacgggca
ctgttactcaaggcactgaccccgttaaaacttattaccagtacactcctgtatcatcaaaagccatgtatgacgcttactggaacggtaaatt
cagagactgcgctttccattctggctttaatgaggatccattcgtttgtgaatatcaaggccaatcgtctgacctgcctcaacctcctgtcaatg
ctggcggcggctctggtggtggttctggtggcggctctgagggtggtggctctgagggtggcggttctgagggtggcggctctgagggaggc
ggttccggtggtggctctggttccggtgattttgattatgaaaagatggcaaacgctaataagggggctatgaccgaaaatgccgatgaaaa
cgcgctacagtctgacgctaaaggcaaacttgattctgtcgctactgattacggtgctgctatcgatggtttcattggtgacgtttccggccttg
ctaatggtaatggtgctactggtgattttgctggctctaattcccaaatggctcaagtcggtgacggtgataattcacctttaatgaataatttcc
gtcaatatttaccttccctccctcaatcggttgaatgtcgcccttttgtctttggcgctggtaaaccatatgaattttctattgattgtgacaaaat
aaacttattccgtggtgtctttgcgtttcttttatatgttgccacctttatgtatgtattttctacgtttgctaacatactgcgtaataaggagtctta
aTACCCGTTTTTTTGGGCTAGAAATAATTTTGTTTAACTTTAAGAAGGAGATATACATATGGCTAGCAAAGGA
GAAGAACTTTTCACTGGAGTTGTCCCAATTCTTGTTGAATTAGATGGTGATGTTAATGGGCACAAATTTTCTG
TCAGTGGAGAGGGTGAAGGTGATGCTACATACGGAAAGCTTACCCTTAAATTTATTTGCACTACTGGAAAAC
TACCTGTTCCATGGCCAACACTTGTCACTACTTTCTCTTATGGTGTTCAATGCTTTTCCCGTTATCCGGATCATA
TGAAACGGCATGACTTTTTCAAGAGTGCCATGCCCGAAGGTTATGTACAGGAACGCACTATATCTTTCAAAG
ATGACGGGAACTACAAGACGCGTGCTGAAGTCAAGTTTGAAGGTGATACCCTTGTTAATCGTATCGAGTTAA
AAGGTATTGATTTTAAAGAAGATGGAAACATTCTCGGACACAAACTCGAGTACAACTATAACTCACACAATGT
ATACATCACGGCAGACAAACAAAAGAATGGAATCAAAGCTAACTTCAAAATTCGCCACAACATTGAAGATG
GATCCGTTCAACTAGCAGACCATTATCAACAAAATACTCCAATTGGCGATGGCCCTGTCCTTTTACCAGACAA
CCATTACCTGTCGACACAATCTGCCCTTTCGAAAGATCCCAACGAAAAGCGTGACCACATGGTCCTTCTTGA

GTTTGTAACTGCTGCTGGGATTACACATGGCATGGATGAGCTCTACAAATACGAT

Plasmid map of M13-lysC

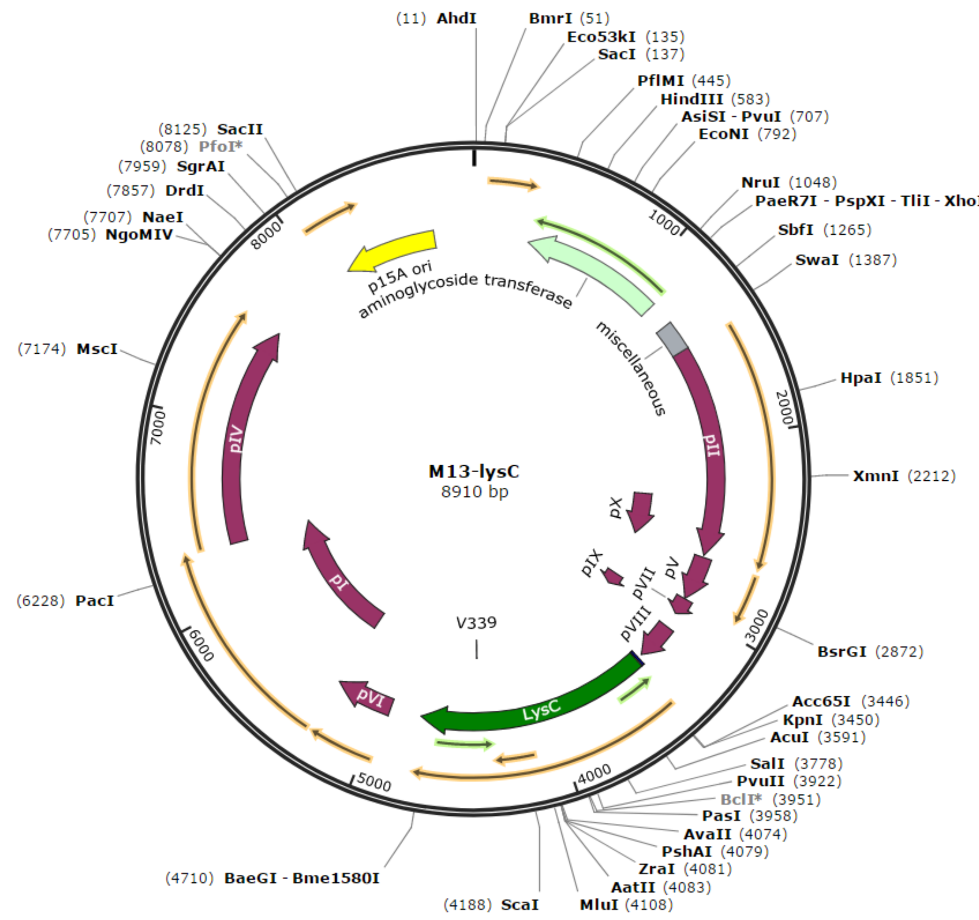

Full sequences of M13-lysC

tgcctgactccccgtcgtgtagataactacgatacgggagggcttaccatctggccccagtgctgcaatgataccgcgagacccacgctcac
cggctccagatttatcagcaataaaccagccagccgattcgagctcgcccggggatcgaccagttggtgattttgaacttttgctttgccacgg
aacggtctgcgttgtcgggaagatgcgtgatctgatccttcaactcagcaaaagttcgatttattcaacaaagccgccgtcccgtcaagtcag
cgtaatgctctgccagtgttacaaccaattaaccaattctgattagaaaaactcatcgagcatcaaatgaaactgcaatttattcatatcagga
ttatcaataccatatttttgaaaaagccgtttctgtaatgaaggagaaaactcaccgaggcagttccataggatggcaagatcctggtatcgg
tctgcgattccgactcgtccaacatcaatacaacctattaatttcccctcgtcaaaaataaggttatcaagtgagaaatcaccatgagtgacga
ctgaatccggtgagaatggcaaaagcttatgcatttctttccagacttgttcaacaggccagccattacgctcgtcatcaaaatcactcgcatc
aaccaaaccgttattcattcgtgattgcgcctgagcgagacgaaatacgcgatcgctgttaaaaggacaattacaaacaggaatcgaatgca
accggcgcaggaacactgccagcgcatcaacaatattttcacctgaatcaggatattcttctaatacctggaatgctgttttcccggggatcgc
agtggtgagtaaccatgcatcatcaggagtacggataaaatgcttgatggtcggaagaggcataaattccgtcagccagtttagtctgaccat
ctcatctgtaacatcattggcaacgctacctttgccatgtttcagaaacaactctggcgcatcgggcttcccatacaatcgatagattgtcgcac
ctgattgcccgacattatcgcgagcccatttatacccatataaatcagcatccatgttggaatttaatcgcggcctcgagcaagacgtttcccgt
tgaatatggctcataacacccccttgtattactgtttatgtaagcagacagttttattgttcatgatgatatatttttatcttgtgcaatgtaacatca
gagattttgaaacacaacgtggctttcccccccccccccctgcaggtctcgggctattcttttgatttataagggattttgccgatttcggcctat
tggttaaaaaatgagctgatttaacaaaaatttaacgcgaattttaacaaaatattaacgtttacaatttaaatatttgcttatacaatcttcctg
tttttggggcttttcttattatcaaccggggtacatatgattgacatgctagttttacgattaccgttcatcgattctcttgtttgctccagactctca
ggcaatgacctgatagcctttgtagacctctcaaaaatagctaccctctccggcatgaatttatcagctagaacggttgaatatcatgttgatg
gtgatttgactgtctccggcctttctcaccctttgaatctttacctacacattactcaggcattgcatttaaaatatatgagggttctaaaaattt

tatccttgcgttgaaataaaggcttctcccgcaaaagtattacagggtcataatgtttttggtacaaccgatttagctttatgctctgaggcttta
ttgcttaattttgctaattctttgccttgcctgtatgatttattggatgttaacgctactactattagtagaattgatgccaccttttcagctcgcgcc
ccaaatgaaaatatagctaaacaggttattgaccatttgcgaaatgtatctaatggtcaaactaaatctactcgttcgcagaattgggaatca
actgttacatggaatgaaacttccagacaccgtactttagttgcatatttaaaacatgttgagctacagcaccagattcagcaattaagctcta
agccatccgcaaaaatgacctcttatcaaaaggagcaattaaaggtactctctaatcctgacctgttggagtttgcttccggtctggttcgcttt
gaagctcgaattaaaacgcgatatttgaagtctttcgggcttcctcttaatctttttgatgcaatccgctttgcttctgactataatagtcagggt
aaagacctgatttttgatttatggtcattctcgttttctgaactgtttaaagcatttgagggggattcaatgaatatttatgacgattccgcagtat
tggacgctatccagtctaaacattttactattaccccctctggcaaaacttcttttgcaaaagcctctcgctattttggtttttatcgtcgtctggta
aacgagggttatgatagtgttgctcttactatgcctcgtaattccttttggcgttatgtatctgcattagttgaatgtggtattcctaaatctcaac
tgatgaatctttctacctgtaataatgttgttccgttagttcgttttattaacgtagatttttcttcccaacgtcctgactggtataatgagccagttc
ttaaaatcgcataaggtaattcacaatgattaaagttgaaattaaaccatctcaagcccaatttactactcgttctggtgtttctcgtcagggca
agccttattcactgaatgagcagctttgttacgttgatttgggtaatgaatatccggttcttgtcaagattactcttgatgaaggtcagccagcct
atgcgcctggtctgtacaccgttcatctgtcctctttcaaagttggtcagttcggttcccttatgattgaccgtctgcgcctcgttccggctaagta
acatggagcaggtcgcggatttcgacacaatttatcaggcgatgatacaaatctccgttgtactttgtttcgcgcttggtataatcgctgggggt
caaagatgagtgttttagtgtattctttcgcctctttcgttttaggttggtgccttcgtagtggcattacgtattttacccgtttaatggaaacttcct
catgaaaaagtctttagtcctcaaagcctctgtagccgttgctaccctcgttccgatgctgtctttcgctgctgagggtgacgatcccgcaaaag
cggcctttaactccctgcaagcctcagcgaccgaatatatcggttatgcgtgggcgatggttgttgtcattgtcggcgcaactatcggtatcaa
gctgtttaagaaattcacctcgaaagcaagctgataaaccgatacaattatactagagaaagaggagaaatactagatgTCTGAAATTG
TTGTCTCCAAATTTGGCGGTACCAGCGTAGCTGATTTTGACGCCATGAACCGCAGCGCTGATATTGTGCTTTC
TGATGCCAACGTGCGTTTAGTTGTCCTCTCGGCTTCTGCTGGTATCACTAATCTGCTGGTCGCTTTAGCTGAA
GGACTGGAACCTGGCGAGCGATTCGAAAAACTCGACGCTATCCGCAACATCCAGTTTGCCATTCTGGAACG
TCTGCGTTACCCGAACGTTATCCGTGAAGAGATTGAACGTCTGCTGGAGAACATTACTGTTCTGGCAGAAGC
GGCGGCGCTGGCAACGTCTCCGGCGCTGACAGATGAGCTGGTCAGCCACGGCGAGCTGATGTCGACCCTG
CTGTTTGTTGAGATCCTGCGCGAACGCGATGTTCAGGCACAGTGGTTTGATGTACGTAAAGTGATGCGTACC
AACGACCGATTTGGTCGTGCAGAGCCAGATATAGCCGCGCTGGCGGAACTGGCCGCGCTGCAGCTGCTCCC
ACGTCTCAATGAAGGCTTAGTGATCACCCAGGGATTTATCGGTAGCGAAAATAAAGGTCGTACAACGACGCT
TGGCCGTGGAGGCAGCGATTATACGGCAGCCTTGCTGGCGGAGGCTTTACACGCATCTCGTGTTGATATCTG
GACCGACGTCCCGGGCATCTACACCACCGATCCACGCGTAGTTTCCGCAGCAAAACGCATTGATGAAATCGC
GTTTGCCGAAGCGGCAGAGATGGCAACTTTTGGTGCAAAAGTACTGCATCCGGCAACGTTGCTACCCGCAG
TACGCAGCGATATCCCGGTCTTTGTCGGCTCCAGCAAAGACCCACGCGCAGGTGGTACGCTGGTGTGCAAT
AAAACTGAAAATCCGCCGCTGTTCCGCGCTCTGGCGCTTCGTCGCAATCAGACTCTGCTCACTTTGCACAGC
CTGAATATGCTGCATTCTCGCGGTTTCCTCGCGGAAGTTTTCGGCATCCTCGCGCGGCATAATATTTCGGTAG
ACTTAATCACCACGTCAGAAGTGAGCGTGGCATTAACCCTTGATACCACCGGTTCAACCTCCACTGGCGATA
CGTTGCTGACGCAATCTCTGCTGATGGAGCTTTCCGCACTGTGTCGGGTGGAGGTGGAAGAAGGTCTGGC
GCTGGTCGCGTTGATTGGCAATGACCTGTCAAAAGCCTGCGGCGTTGGCAAAGAGGTATTCGGCGTACTGG
AACCGTTCAACATTCGCATGATTTGTTATGGCGCATCCAGCCATAACCTGTGCTTCCTGGTGCCCGGCGAAGA
TGCCGAGCAGGTGGTGCAAAAACTGCATAGTAATTTGTTTGAGtaactccctcaatcggttgaatgtcgcccttttgtcttt
ggcgctggtaaaccatatgaattttctattgattgtgacaaaataaacttattccgtggtgtctttgcgtttcttttatatgttgccacctttatgta
tgtattttctacgtttgctaacatactgcgtaataaggagtcttaatcatgccagttcttttgggtattccgttattattgcgtttcctcggtttccttc
tggtaactttgttcggctatctgcttacttttcttaaaaagggcttcggtaagatagctattgctatttcattgtttcttgctcttattattgggctta
actcaattcttgtgggttatctctctgatattagcgctcaattaccctctgactttgttcagggtgttcagttaattctcccgtctaatgcgcttccc
tgtttttatgttattctctctgtaaaggctgctattttcatttttgacgttaaacaaaaaatcgtttcttatttggattgggataaataatatggctgt
ttattttgtaactggcaaattaggctctggaaagacgctcgttagcgttggtaagattcaggataaaattgtagctgggtgcaaaatagcaac
taatcttgatttaaggcttcaaaacctcccgcaagtcgggaggttcgctaaaacgcctcgcgttcttagaataccggataagccttctatatct

gatttgcttgctattgggcgcggtaatgattcctacgatgaaaataaaaacggcttgcttgttctcgatgagtgcggtacttggtttaatacccg
ttcttggaatgataaggaaagacagccgattattgattggtttctacatgctcgtaaattaggatgggatattatttttcttgttcaggacttatct
attgttgataaacaggcgcgttctgcattagctgaacatgttgtttattgtcgtcgtctggacagaattactttaccttttgtcggtactttatattc
tcttattactggctcgaaaatgcctctgcctaaattacatgttggcgttgttaaatatggcgattctcaattaagccctactgttgagcgttggct
ttatactggtaagaatttgtataacgcatatgatactaaacaggctttttctagtaattatgattccggtgtttattcttatttaacgccttattat
cacacggtcggtatttcaaaccattaaatttaggtcagaagatgaaattaactaaaatatatttgaaaaagttttctcgcgttctttgtcttgcg
attggatttgcatcagcatttacatatagttatataacccaacctaagccggaggttaaaaaggtagtctctcagacctatgattttgataaatt
cactattgactcttctcagcgtcttaatctaagctatcgctatgttttcaaggattctaagggaaaattaattaatagcgacgatttacagaagc
aaggttattcactcacatatattgatttatgtactgtttccattaaaaaaggtaattcaaatgaaattgttaaatgtaattaattttgttttcttgat
gtttgtttcatcatcttcttttgctcaggtaattgaaatgaataattcgcctctgcgcgattttgtaacttggtattcaaagcaatcaggcgaatcc
gttattgtttctcccgatgtaaaaggtactgttactgtatattcatctgacgttaaacctgaaaatctacgcaatttctttatttctgttttacgtgc
aaataattttgatatggtaggttctaacccttccattattcagaagtataatccaaacaatcaggattatattgatgaattgccatcatctgata
atcaggaatatgatgataattccgctccttctggtggtttctttgttccgcaaaatgataatgttactcaaacttttaaaattaataacgttcggg
caaaggatttaatacgagttgtcgaattgtttgtaaagtctaatacttctaaatcctcaaatgtattatctattgacggctctaatctattagttgt
tagtgctcctaaagatattttagataaccttcctcaattcctttcaactgttgatttgccaactgaccagatattgattgagggtttgatatttgag
gttcagcaaggtgatgctttagatttttcatttgctgctggctctcagcgtggcactgttgcaggcggtgttaatactgaccgcctcacctctgttt
tatcttctgctggtggttcgttcggtatttttaatggcgatgttttagggctatcagttcgcgcattaaagactaatagccattcaaaaatattgtc
tgtgccacgtattcttacgctttcaggtcagaagggttctatctctgttggccagaatgtcccttttattactggtcgtgtgactggtgaatctgcc
aatgtaaataatccatttcagacgattgagcgtcaaaatgtaggtatttccatgagcgtttttcctgttgcaatggctggcggtaatattgttctg
gatattaccagcaaggccgatagtttgagttcttctactcaggcaagtgatgttattactaatcaaagaagtattgctacaacggttaatttgcg
tgatggacagactcttttactcggtggcctcactgattataaaaacacttctcaggattctggcgtaccgttcctgtctaaaatccctttaatcgg
cctcctgtttagctcccgctctgattctaacgaggaaagcacgttatacgtgctcgtcaaagcaaccatagtacgcgccctgtagcggcgcatt
aagcgcggcgggtgtggtggttacgcgcagcgtgaccgctacacttgccagcgccctagcgcccgctcctttcgctttcttcccttcctttctcg
ccacgttcgccggctttccccgtcaagctctaaatcgggggctccctttagggttccgatttagtgctttacggcacctcgaccccaaaaaactt
gatttgggtgatggttcacgtagtgggccatcgccctgatagacggtttttcgccctttgacgttggagtccacgttctttaatagtggactcttg
ttccaaactggaacaacactcaaccctatctcgggacggatcgcttcatgtggcaggagaaaaaaggctgcaccggtgcgtcagcagaata
tgtgatacaggatatattccgcttcctcgctcactgactcgctacgctcggtcgttcgactgcggcgagcggaaatggcttacgaacggggcg
gagatttcctggaagatgccaggaagatacttaacagggaagtgagagggccgcggcaaagccgtttttccataggctccgcccccctgac
aagcatcacgaaatctgacgctcaaatcagtggtggcgaaacccgacaggactataaagataccaggcgtttccccctggcggctccctcgt
gcgctctcctgttcctgcctttcggtttaccggtgtcattccgctgttatggccgcgtttgtctcattccacgcctgacactcagttccgggtaggc
agttcgctccaagctggactgtatgcacgaaccccccgttcagtccgaccgctgcgccttatccggtaactatcgtcttgagtccaacccggaa
agacatgcaaaagcaccactggcagcagccactggtaattgatttagaggagttagtcttgaagtcatgcgccggttaaggctaaactgaaa
ggacaagttttggtgactgcgctcctccaagccagttacctcggttcaaagagttggtagctcagagaaccttcgaaaaaccgccctgcaag
gcggttttttcgttttcagagcaagagattacgcgcagaccaaaacgatctcaagaagatcatcttattaaggggtctgacgctcagtggaac
gaaaactcacgttaagggattttggtcatgagattatcaaaaaggatcttcacctagatccttttaaattaaaaatgaagttttaaatcaatct
aaagtatatatgagtaaacttggtctgacagttaccaatgcttaatcagtgaggcacctatctcagcgatctgtctatttcgttcatccatagt
